# A dynamic prediction model of landslide displacement based on VMD–SSO–LSTM approach

**DOI:** 10.1038/s41598-024-59517-2

**Published:** 2024-04-22

**Authors:** Haiying Wang, Yang Ao, Chenguang Wang, Yingzhi Zhang, Xiaofeng Zhang

**Affiliations:** 1 School of Construction Machinery, Chang’an University, Xi’an, 710064 China; 2 Shaanxi Transportation Holding Group Co., Ltd., Xi’an, 710075 China

**Keywords:** Civil engineering, Computer science, Natural hazards

## Abstract

Addressing the limitations of existing landslide displacement prediction models in capturing the dynamic characteristics of data changes, this study introduces a novel dynamic displacement prediction model for landslides. The proposed method combines Variational Mode Decomposition (VMD) with Sparrow Search Optimization (SSO) and Long Short-Term Memory (LSTM) techniques to formulate a comprehensive VMD–SSO–LSTM model. Through the application of VMD, the method dissects cumulative displacement and rainfall data, thereby extracting distinct components such as trend, periodicity, and fluctuation components for displacement, as well as low-frequency and high-frequency components for rainfall. Furthermore, leveraging Gray Correlational Analysis, the interrelationships between the periodic component of displacement and the low-frequency component of rainfall, as well as the fluctuation component of displacement and the high-frequency component of rainfall, are established. Building upon this foundation, the SSO–LSTM model dynamically predicts the interrelated displacement components, synthesizing the predicted values of each component to generate real-time dynamic forecasts. Simulation results underscore the effectiveness of the proposed VMD–SSO–LSTM model, indicating root-mean-square error (RMSE) and mean absolute percentage error (MAPE) values of 1.2329 mm and 0.1624%, respectively, along with a goodness of fit (R^2^) of 0.9969. In comparison to both back propagation (BP) prediction model and LSTM prediction model, the VMD–SSO–LSTM model exhibits heightened predictive accuracy.

## Introduction

Landslides rank among the most significant geological disasters, with their occurrence leading to profound destructive impacts on both the ecological environment and the lives and properties of communities. The prediction of landslide deformation has consistently posed a challenging research endeavor^1^. Rational construction of a predictive model for landslide displacement stands as a pivotal technology for achieving effective monitoring and early warning of landslide geological hazards. Timely alerts possess the potential to mitigate, or even avert, casualties and property losses, bearing notable theoretical and practical significance^2^.

Landslide displacement curves generally manifest as non-stationary time series. Typically, historical data is employed for constructing geometric models^3^ or employing gray models^4^ to forecast landslide displacement, yet these methods tend to yield lower predictive accuracy. Some scholars have enhanced the predictive accuracy of landslide displacement through time series decomposition, utilizing different prediction methods based on the characteristics of decomposition components^5–13^. Currently, two primary methods are employed for decomposing cumulative landslide displacement time series. The first method involves mathematical techniques such as moving averages method^5,6^ and exponential smoothing method^11^, which facilitate the extraction of physically meaningful trend and periodic components from cumulative displacement. The second method involves decomposing cumulative displacement based on signal separation theory. For instance, Ma et al.^12^ used DB4 wavelet transform to decompose the displacement sequence into trend term displacement and periodic term displacement, which effectively reduced the noise of the original data. However, wavelet analysis often faces challenges in determining appropriate wavelet bases. Shihabudheen et al.^14^ used empirical mode decomposition (EMD) to decompose the landslide displacement data into three IMFs and one residual, and then predicted the decomposed IMF/residual respectively. Finally, the cumulative displacement is obtained by summarizing the prediction results, which effectively improves the prediction accuracy. However, EMD decomposition encounters mode mixing issues when abrupt temporal changes are present in displacement signals. To address the mode mixing problem in EMD decomposition, Du et al.^8^ introduced a method that integrates EMD with white noise, termed Ensemble Empirical Mode Decomposition (EEMD). This approach tailor’s data decomposition according to the intrinsic time scale characteristics of monitoring data, making it suitable for trend extraction and decomposition. Niu et al.^9^, utilizing EEMD, successfully decomposed Three Gorges reservoir landslide displacement into trend and periodic components, resulting in favorable predictive outcomes. Zhang et al.^10^ employed VMD to adaptively decompose landslide displacement time series signals, overcoming mode mixing issues observed in EMD. Notably, they achieved commendable predictive results for step-like landslide displacement. Additionally, Gao et al.^13^ utilized VMD to decompose cumulative landslide displacement into trend and seasonal fluctuation sequences, enhancing the predictive accuracy of environmentally influenced landslide displacement.

The displacement prediction models for landslides can be categorized into two types: static prediction models and dynamic prediction models. Yan et al.^15^ added environmental monitoring variables such as the free slope gradient, slide surface slope gradient, and soil texture of the mountain mass to the construction of the static BP model, which significantly improved the accuracy and stability of the landslide warning model. Zhang et al.^16^ used the improved WCA–BPNN model to study the landslide in Langshuwan, which better solved the shortcomings of slow BP convergence, easy to fall into the local optimal solution and poor dynamic characteristics. Shang et al.^11^ utilized a static prediction model based on Hybrid Kernel Function Support Vector Machine Regression (SA-SVR) to forecast the periodic displacement of landslides. Balogun et al.^17^ fine-tuned the parameters of the SVR model through a variety of intelligent algorithms such as gray Wolf and cuckoo, effectively improving the accuracy and stability of the model prediction. Wang et al.^18^ proposed a Particle Swarm Optimization and Least Squares Support Vector Machine (PSO–LSSVM) static prediction model to predict the fluctuating displacement of landslides. However, these static models overlook the fact that landslide evolution is a complex dynamic system and they fail to extract relevant features from displacement time series. To address these limitations, scholars have turned to the use of LSTM, a dynamic prediction model that captures the time series characteristics of displacement, in landslide displacement prediction. Li et al.^19^ employed LSTM for the prediction of Xintan landslide, achieving improved accuracy in capturing the dynamic behavior of landslide displacement. Hamedi et al.^20^ selected 12 landslide influencing factors to construct a LSTM algorithm to make landslide susceptibility regionalization of Adabirson, Iran, and the prediction results had a small error and high precision. Tengtrairat et al.^21^ combined four static factors (land cover, soil properties, elevation and slope) and one dynamic factor (precipitation) to build Bi-LSTM algorithm to study landslides in Chiang Rai, Thailand, improving the algorithm model's ability to capture dynamic characteristics of landslides. Wang et al.^22^ utilized Gray Correlation Degree to select influencing factors for Bazhimen landslide and built a CNN-LSTM model on this basis, effectively enhancing the model's generalization capability and predictive accuracy. In the aforementioned studies, the predictive accuracy of LSTM models relies on the optimization and selection of model parameters. To enhance predictive accuracy, it is necessary to optimize the parameters of the LSTM model using parameter optimization algorithms. The Sparrow Search Algorithm^23^, known for its high stability and strong global search capability, can rapidly identify optimal values. Employing SSA for optimizing LSTM's hyper-parameters can effectively enhance the model's predictive accuracy^24^.

In summary, EMD can effectively decompose displacement into distinct feature components, but it is susceptible to mode mixing. The use of LSTM dynamic prediction model for displacement time series signals yields favorable predictive outcomes, yet determining model parameters poses challenges. Therefore, exploring effective decomposition of landslide displacement signals and optimizing parameters for the dynamic LSTM prediction model holds crucial significance for enhancing predictive accuracy. This paper introduces an SSO–LSTM dynamic landslide displacement prediction model based on VMD. VMD is employed to decompose cumulative displacement and rainfall, followed by conducting Gray Correlation Analysis on the decomposed components. The correlated displacement components are then utilized as inputs for the dynamic prediction model. Leveraging the strengths of the SSO algorithm, characterized by high stability and strong global search capability, the SSO–LSTM model is constructed for dynamic landslide displacement prediction. This model forecasts the evolution trend of landslides, contributing to the improvement of landslide risk management practices.

## Overview of the study area

### Study area

**Figure 1:**
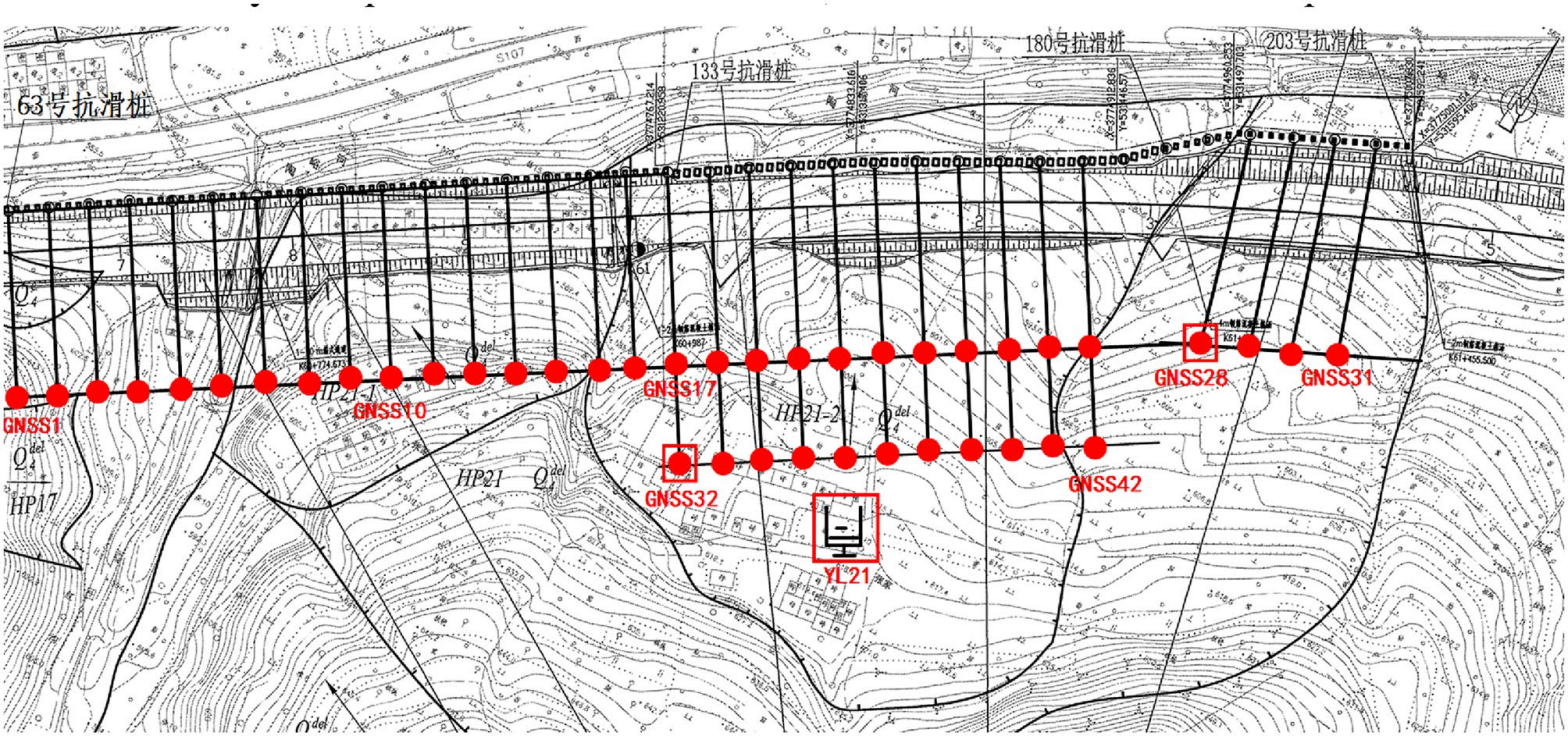
Plan view of the monitoring point layout for the HP21 landslide: GNSS displacement points: GNSS1 to GNSS42. Rain gauge: YL21 (Name of software: ArcGIS 10.5, URL: https://www.esri.com/en-us/arcgis/about-arcgis/overview).

The case study in this research focuses on landslide body HP21, which spans a total length of 643 m, with a slope height of 40.3 m and a slope gradient of 1:1. The geological and topographical survey of the landslide area reveals an uneven terrain and significant incision of the valleys. The plan view of the HP21 landslide body along with the layout of measurement sensors was shown in Fig. 1. The monitoring network for the landslide body comprises one rainfall sensor, 42 GNSS cumulative displacement sensors.

### Data sources

**Figure 2:**
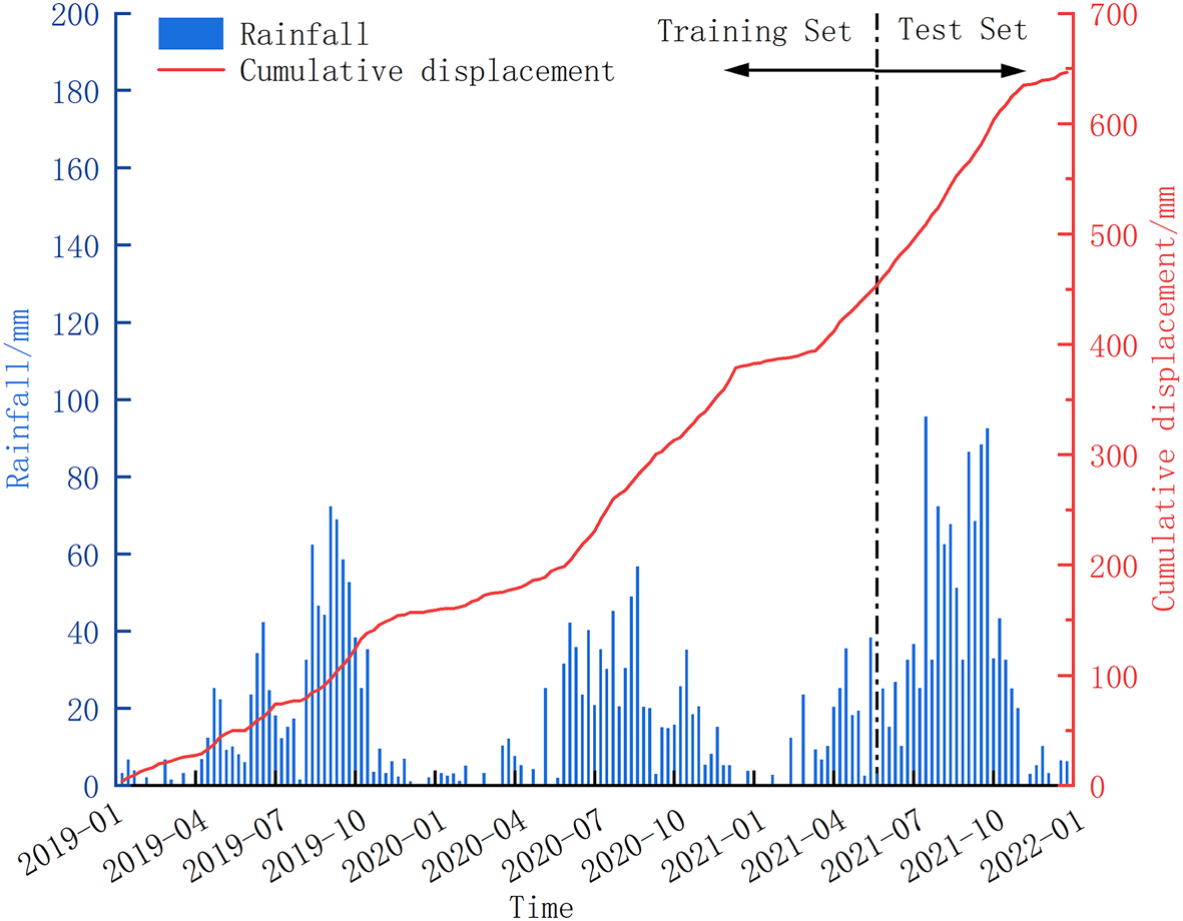
GNSS32 Cumulative displacement and weekly rainfall data.

The HP21 monitoring system was installed in October 2018 with a data collection frequency of 1 day per event. We collected 3 years of monitoring data on rainfall sensor (YL21) and displacement sensors (GNSS32 and GNSS28) from 2019 to 2021 for training and validating prediction models. GNSS32, located in the middle and lower part of the landslide, was selected for detailed analysis, while GNSS28, located in the upper right edge, was chosen for comparative analysis. The selected monitoring data are illustrated in Fig. 2. The data sets were divided into a ratio of 8:2, with monitoring data from January 5, 2019, to May 28, 2021, used as the training set, and monitoring data from May 29, 2021, to December 25, 2021, used as the testing set.

As depicted in Fig. 2, the precipitation influencing the landslide is primarily concentrated from June to October, with September experiencing the peak of rainfall intensity. With increasing rainfall, when the rainfall reaches a certain intensity, the cumulative displacement curve will produce an inflection point and then transition to another phase. Landslide displacement is accompanied by heavy rainfall in the three stages of rapid deformation, and the rapid deformation of landslide displacement lags slightly behind the heavy rainfall. The duration of this lag is typically intertwined with the characteristics of the precipitation, the internal permeability of the soil layers within the landslide, and other contributing factors. In summary, precipitation appears to be the primary catalyst initiating the movement of the HP21 landslide body. Consequently, in the subsequent formulation of measurement models, careful consideration must be given to understanding its effect on landslide body displacement.

## Methodology

### The proposed VMD–SSO–LSTM prediction method

**Figure 3:**
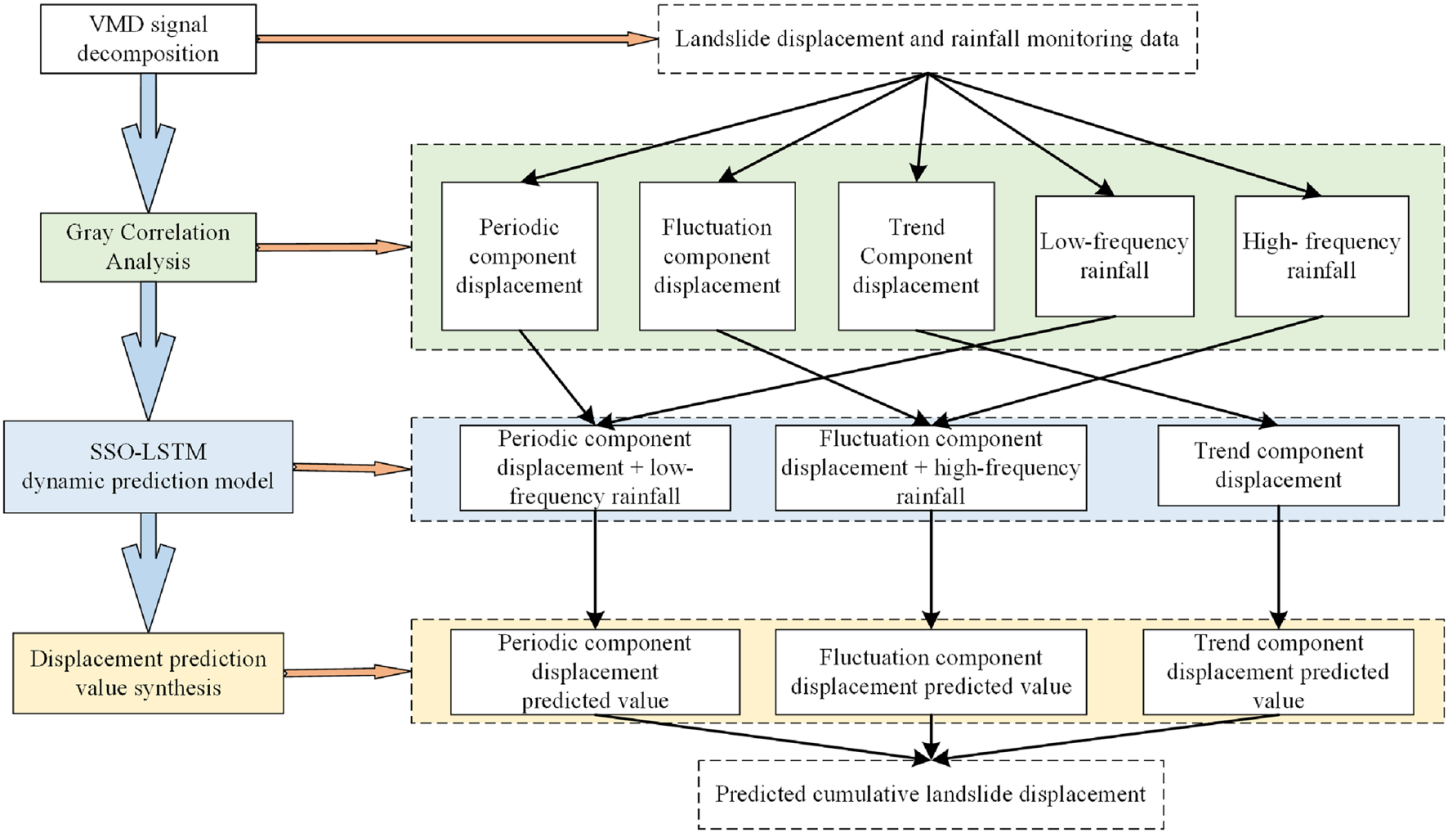
The VMD–SSO–LSTM dynamic prediction method for landslide displacement.

The proposed VMD–SSO–LSTM dynamic landslide displacement prediction method consists of four main components: VMD signal decomposition, component correlation using Gray Correlation Analysis, SSO–LSTM prediction of correlated displacement signals, and synthesis of cumulative displacement. The detailed process is illustrated in Fig. 3.

VMD were adopted to decompose the time series of cumulative landslide displacement and rainfall. The decomposed displacement included trend, periodic, and fluctuation components. The decomposed rainfall included high-frequency and low-frequency components. Gray Correlation analysis is employed to establish associations between displacement and rainfall components by matching their correlation features. LSTM rolling prediction method is used to predict the trend component displacement sub-sequence. The SSO–LSTM model was used to predict the periodicity and fluctuation sub-sequences. Cumulative displacement synthesis involves combining predicted values of various displacement sub-sequences.

### VMD decomposition of displacement and rainfall sequences

VMD is an adaptive and fully non-recursive method for mode variational and signal processing^25^. The specific steps are as follows:

Step 1: Constructing the constrained variational problem.

Assuming that the original time series signal *f* is decomposed into *k* modal components *u*_k_(t) with bandwidth. The center frequency of each intrinsic Mode function (IMF) component is denoted as *ω*_k_. The constraint applied is that the sum of the all-modal components equals the original signal. Consequently, the constrained variational expression is as follows:

1 $$\left\{ \begin{gathered} \mathop {\min }\limits_{{\{ u_{k} \} ,\;\{ \omega_{k} \} }} \left\{ {\sum\limits_{k = 1} {\left\| {\partial_{t} [\delta (t) + \frac{j}{\pi t}*u_{k} (t)]e^{{ - j\omega_{k} t}} } \right\|}_{2}^{2} } \right\} \hfill \\ s.t.\;\;\sum\limits_{k = 1}^{K} {u_{k} = f} \hfill \\ \end{gathered} \right.$$

where $\{ u_{k} \} = \{ u_{1} , \ldots ,u_{k} \}$ represent all IMF components, $\{ \omega_{k} \} = \{ \omega_{1} , \ldots ,\omega_{k} \}$ signify the central frequency of each component. *∂*_*t*_ represents the partial derivative with respect to *t*, *δ(t)*represents the Dirac delta function, * represents the convolution.

Step 2: Transformation into an unconstrained variational problem.

Lagrange multipliers *λ* and quadratic penalty factors* α* are introduced to convert the constrained variational problem into an unconstrained variational problem. The extended Lagrangian expression is given by:

2 $$\begin{aligned} L(\{ u_{k} \} ,\;\{ \omega_{k} \} ,\lambda ) & = \alpha \sum\limits_{k = 1}^{{\phantom{a}}} {\left\| {\partial_{t} \left[ {\left( {\delta (t) + \frac{j}{\pi t}} \right)*u_{k} (t)} \right]e^{{ - j\omega_{k} t}} } \right\|}_{2}^{2} + \left\| {f(t) - \sum\limits_{k = 1}^{{\phantom{a}}} {u_{k} (t)} } \right\|_{2}^{2} \\ & \quad + \left\langle {\lambda (t),f(t) - \sum\limits_{k = 1}^{{\phantom{a}}} {u_{k} (t)} } \right\rangle \\ \end{aligned}$$

Step 3: Solving the saddle point of the Unconstrained Variational Model.

By utilizing the alternating direction method of multipliers (ADMM), the modal components and their central frequencies are iteratively updated to reach the saddle point of the unconstrained variational model. This yields the optimal solution to the constrained variational problem:

3 $$\hat{u}_{k}^{n + 1} \;(\omega ) = \frac{{\hat{f}(\omega ) - \sum\limits_{i \ne k} {\hat{u}_{i} (\omega )} + \hat{\lambda }(\omega )/2}}{{1 + 2\alpha (\omega - \omega_{k} )^{2} }}$$

where ω represents the central frequency of each component, $\hat{u}_{k}^{n + 1} (\omega )$, $\hat{f}(\omega )$, $\hat{\lambda }(\omega )$ corresponds to the Fourier transformation of $u_{k}^{n + 1} (t)$, f(t), λ(t) respectively.

Step 4: Re-estimation of center frequency for each IMF.

$\hat{u}_{k}^{n + 1} (\omega )$ is the residual of $\hat{f}(\omega ) - \sum\nolimits_{i \ne k} {\hat{u}_{k} (\omega )}$ after Wiener filtering. The center frequencies are re-estimated based on the centroid of the power spectra for each IMFs. The updates for $\hat{u}_{k}^{n + 1}$ using formula (3), and the updates for *ω*_k_, and $\hat{\lambda }$ are as follows:

4 $$\omega_{k}^{n + 1} = \frac{{\int_{0}^{\infty } {\omega \left| {u_{k}^{n + 1} (\omega )} \right|}^{2} d\omega }}{{\int_{0}^{\infty } {\left| {u_{k}^{n + 1} (\omega )} \right|}^{2} d\omega }}$$

5 $$\hat{\lambda }^{n + 1} (\omega ) = \hat{\lambda }^{n} (\omega ) + \lambda \left( {\hat{f}(\omega ) - \sum\limits_{k} {\hat{u}_{k}^{n + 1} (\omega )} } \right)$$

Step 5: Iteration stopping criteria.

The iteration process for solving the central frequencies of each modal component is halted when the stopping condition is met, as defined by formula (6).

6 $$\frac{{\sum\nolimits_{k = 1}^{K} {\left\| {\hat{u}_{k}^{n + 1} - \hat{u}_{k}^{n} } \right\|}_{2}^{2} }}{{\left\| {\hat{u}_{k}^{n} } \right\|_{2}^{2} }} < \varepsilon_{t}$$

### Gray correlation analysis of VMD decomposed IMFs

Gray Correlation Analysis is a method for quantitatively describing and comparing the development and changing trends a system. It involves analyzing and calculating numerical values of specified indicator series and several comparative series. The correlation closeness is determined based on similarity of their geometric shapes. The rainfall studied in this paper is an important factor affecting landslide displacement, but the data of landslide displacement cannot establish a direct mathematical relationship with rainfall. Therefore, the mathematical relationship between sub-displacement and sub-rainfall is obtained by grey correlation analysis of IMFs derived from the decomposition of cumulative displacement and rainfall, which can be used to improve the prediction accuracy of landslide displacement in subsequent studies.

### SSO–LSTM landslide dynamic displacement prediction model

**Figure 4:**
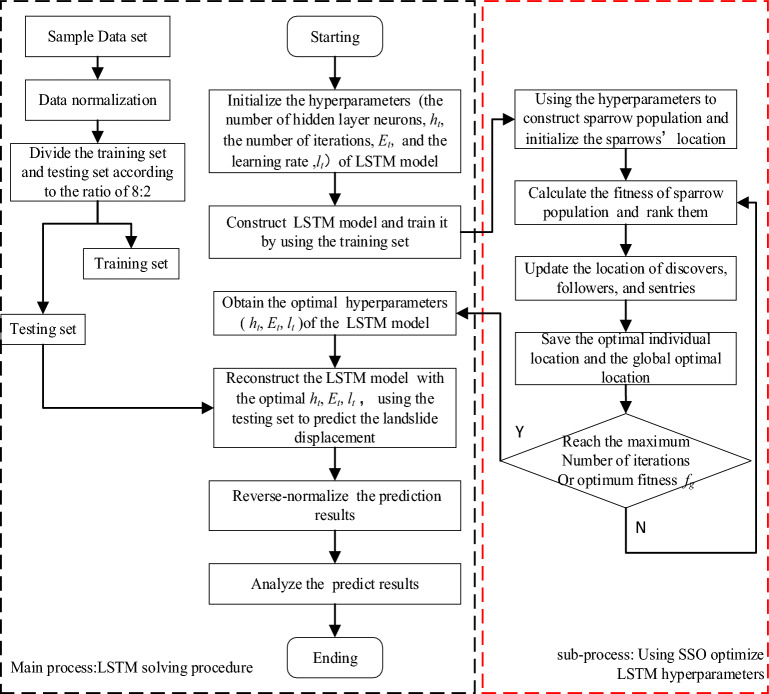
Flowchart of SSO–LSTM dynamic prediction model of landslide.

LSTM is composed of three main gate components including input gate, *i*, forgetting gate, *f*, and output gate, *y,* on the basis of RNN. The model solves the problem of gradient disappearance and gradient explosion in the long sequence training process of traditional RNN model. It has very good performance ability for forecasting long time series data. However, the prediction accuracy of the LSTM model is greatly affected by its hyper-parameters such as the number of hidden layer neurons (*h*_*t*_), the number of iterations (*E*_*t*_), and the learning rate (*l*_*t*_). These hyper-parameters are set in a value range interval. Traditional hyper-parameter adjustment methods often rely on experience and trial and error, which is difficult to ensure the model performance. The SSO algorithm compares the process of parameter optimization to that of sparrow population searching for food. It has fast convergence and strong optimization ability^23^. Therefore, the SSO–LSTM landslide displacement dynamic prediction model is proposed in this paper, as shown in Fig. 4.

The SSO method are used to optimize the hyper-parameters of the LSTM model. With the global search capability of SSA algorithm, LSTM model can quickly and stably converge to the global optimal solution, thus significantly improving the performance and prediction accuracy of the model. The specific steps involved in SSO-optimized LSTM model solving process are outlined as follows:

Step 1: According the regions of the LSTM key hyper-parameters *h*_*t*_, *E*_*t*_, and *l*_*t*_, initialize the population and related parameters such as dimensionality of the problem (*j*), maximum number of iterations (*T*), population size (*N*), warning threshold (*R*_2_), and safety threshold (*S*). Randomly generate initial sparrow positions to form the initial population.

Step 2: The predicted value of the LSTM model and the root-mean-square value of the sample data set were selected to determine the fitness value of each sparrow, ranking them and selecting the best and worst sparrow individuals at present. A higher fitness value indicates a better solution at that position.

Step 3: Update the discoverers’ position

7 $$X_{i,j}^{t + 1} = \left\{ \begin{gathered} X_{i,j}^{t + 1} \cdot \exp \left( { - \frac{i}{a \cdot T}} \right),\;\;R_{2} < S \hfill \\ X_{i,j}^{t} + Q \cdot L,\;R_{2} \ge S \hfill \\ \end{gathered} \right.$$

where *x*_*i*,*j*_ represents the sparrow’s position *i* in the *j*-th dimension, t indicates the current of iterations. a is a (0,1] random number. Q represents a random number with a positive distribution,*** L*** is a 1 × d matrix with all 1 element. *R*_2_ and *S* represent the warning threshold and the safety threshold, respectively. When *R* < *S,* it indicates a state of safety, prompting the discoverer to continue the search. Conversely, when *R*_2_ ≥ *S*, signifying a state of danger, the population must promptly relocate to a secure area.

Step 4: Update the position of followers: For each sparrow, further update its position using information from the current position, the best position found so far, and potentially the worst position. Utilize a matrix of elements to adjust the direction of the position change.

8 $$X_{i,j}^{t + 1} = \left\{ \begin{gathered} Q \cdot \exp \left( { - \frac{{X_{worst}^{t} - X_{i,j}^{t} }}{{i^{2} }}} \right),\;\;i < \frac{n}{2} \hfill \\ X_{P}^{t + 1} + \left| {X_{i,j}^{t} - X_{P}^{t + 1} } \right| \cdot A^{ + } \cdot L,\;\;i \ge \frac{n}{2} \hfill \\ \end{gathered} \right.$$

where $X_{P}^{t}$ and $X_{worst}^{t}$ are the optimal position and the worst position in the *t*-th generation iteration respectively. *A* is a *1* × *d* matrix with the element values is − 1 or 1, *A*^+^ = *A*^+^(*AA*^+^)^−1^.

Step 5: Defining the sentries’ population and updating position through iterative optimizations. A subset of sentries, ranging from 10 to 20% of the population, is designated as having awareness of danger. The initial positions of the sentries are randomly generated. The formula for updating the positions of the sentries with awareness of danger is as follows:

9 $$X_{i,j}^{t + 1} = \left\{ \begin{gathered} X_{best}^{t} + \beta \left| {X_{i,j}^{t} - X_{best}^{t} } \right|,\;\;f_{i} > f_{g} \hfill \\ X_{i,j}^{t} + K\frac{{\left| {X_{i,j}^{t} - X_{best}^{t} } \right|}}{{(f_{i} - f_{w} ) + \varepsilon }},\;\;f_{i} = f_{g} \hfill \\ \end{gathered} \right.$$

where *X*_*best*_ represents the current global optimal position. *k, β* depict the step control parameters, and *k* is a random number belonging to [− 1,1], while *β* is a random number with a positive-taira distribution obeying a mean of 0 and a variance of 1. *f*_*i*_, *f*_*w*_ and *f*_*g*_ indicate the current, the worst and best fitness values of the sentries, respectively. When *f*_*i*_ > *f*_*g*_, it indicates that the sentry is at the edge of the population and is vulnerable to harm. Conversely, when *f*_*i*_ = *f*_*g*_, it indicates that the sentry is in the middle of the population and is aware of the danger and needs to get closer to the other sparrows. *ε* is the smallest constant, *ε* > 0.

Step 6: Determine whether it finds the best fitness fg or reaches the maximum number of iterations. If so, the optimized parameters are obtained. If it is, proceed to Step 2.

Step 7: The optimal hyper-parameters value of *h*_*t*_, *E*_*t*_, and *l*_*t*_ are obtained and the LSTM model is reconstructed with the optimal parameters.

## Results

### VMD decomposition and reconstruction

The Augmented Dickey Fuller (ADF) test is a traditional approach to examining whether a time series is stationary. This paper employs the ADF test to ascertain if the time series derived from VMD has undergone excessive decomposition, and consequently, to determine the number of mode decomposition, *K*. Table 1 shows the ADF test values for the different IMFs under different values of *K*.

**Table 1:** ADF test values for IMFs under different *K* values.

	K = 2	K = 3	K = 4	K = 5	K = 6	K = 7
IMF1	− 5.826	− 6.157	− 4.048	− 2.805	− 1.073	− 3.738
IMF2	− 5.932	− 6.102	− 5.091	− 3.142	− **2.642**	− **3.371**
IMF3		− 6.656	− 4.838	− 4.035	− **8.873**	− **8.393**
IMF4			− 4.375	− 3.252	− 5.323	− 4.643
IMF5				− 2.535	− 5.354	− 6.353
IMF6					− 5.265	− 5.239
IMF7						− 5.197

Significant values are in bold.

**Figure 5:**
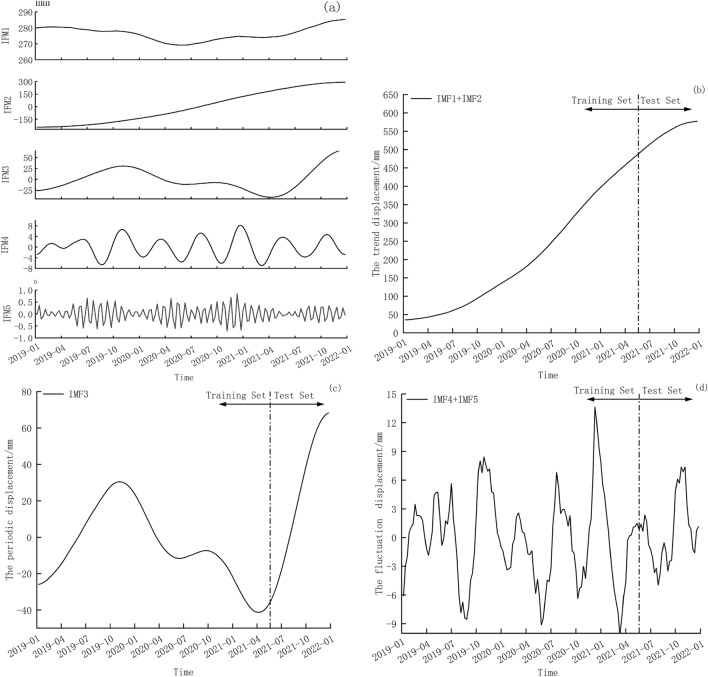
(**a**) The VMD decomposition of landslides cumulative displacement, (**b**) the reconstructed trend component displacement (IMF1 + IMF2), (**c**) the periodic component displacement (IMF3) and (**d**) the reconstructed fluctuation component displacement (IMF4 + IMF5).

Upon analyzing the computational results from Table 1, it is discerned that, when *K* = 6, the ADF test values for IMF3 exhibit a marked reduction compared to those of IMF2. Hence, it is postulated that a phenomenon of excessive decomposition commences in VMD. Consequently, the optimal decomposition count is determined to be *K* = 5. A penalty parameter, *α,* is set at 2000 based on empirical wisdom and a convergence termination criterion, *ε*, is set at 10^−7^. The resulting cumulative displacement of the VMD decomposition's IMFs are shown in Fig. 5a.

It is evident that the IMF1 and IMF2 show a slow upward trend, and have minimal fluctuations. Therefore, the IMF1 and IMF2 are combined to obtain the trend displacement component, as shown in Fig. 5b. The IMF3 fluctuates slightly and is close to the periodic rainfall trend. Therefore, IMF3 is used as the periodic displacement component, as shown in Fig. 5c. The IMF4 and IMF5 are relatively small with strong fluctuation. Therefore, the two sets are reconstructed to obtain the fluctuation displacement component, as shown in Fig. 5d.

**Figure 6:**
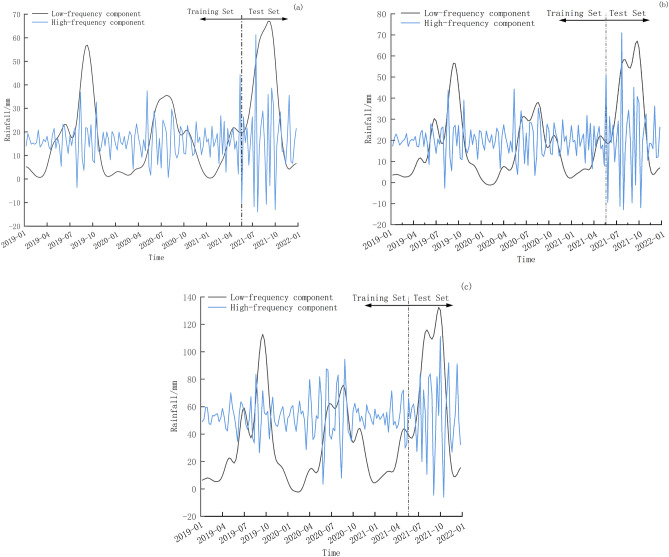
The rainfall VMD decomposition of (**a**) the current week, (**b**) the previous week and (**c**) the previous 2 weeks.

Due to the delayed impact of precipitation on landslide displacement, we have chosen to compile a repository of rainfall factors, comprising the rainfall of the current week, the rainfall of the previous week, and the accumulated rainfall of the previous 2 weeks. During the rainy season, rainfall exhibits distinct periodicity and randomness^26^. Through VMD decomposition, the rainfall factor is disintegrated into high-frequency and low-frequency components, as shown in Fig. 6.

### Gray correlation analysis

The results of gray correlation analysis for the reconstructed components of landslide displacement and the graded components of rainfall are presented in Table 2. Based on the selection criterion of a gray correlation *r*_*i*_ ≥ 0.7, it can be seen that: (1) The correlation between the trend component displacement and the rainfall factor is relatively low, indicating that the trend component displacement is mainly influenced by intrinsic factors of landslide movement and is independent of rainfall. (2) The correlation between the periodic component displacement and the rainfall low-frequency component is approximately 0.92, Therefore, the rainfall low-frequency component serves as a characteristic factor for predicting the periodic displacement component. (3) The correlation between the fluctuation component displacement and the high-frequency component of rainfall is much higher than the selection criterion of 0.7. Hence, high frequency components serve as a discriminator for predicting fluctuating displacement components.

**Table 2:** Gray correlation analysis results of landslide displacement components and rainfall components.

Gray correlation *r*_*i*_		Landslide displacement VMD component		
		Trend component	Periodic component	Fluctuation component
Rainfall of the current week	Low-frequency	0.652	**0.931**	0.681
	High-frequency	0.589	0.685	**0.941**
Rainfall of the previous week	Low-frequency	0.682	**0.921**	0.679
	High-frequency	0.597	0.671	**0.859**
Accumulated rainfall of the previous 2 weeks	Low-frequency	0.645	**0.928**	0.712
	High-frequency	0.608	0.645	**0.933**

Significant values are in bold.

### Prediction results and comparison

The sample data sets of trend components, period components, and fluctuation components were used to separately train and test the proposed SSO–LSTM model, the traditional LSTM model, and the traditional BP model. The testing results were compared to the actual monitoring values to validate the superiority of the proposed SSO–LSTM prediction model. Evaluation metrics including Root Mean Square Error (RMSE), Mean Absolute Percentage Error (MAPE) and Goodness of Fit (R^2^) were employed to assess the accuracy of the three models. Smaller RMSE and MAPE values indicated lower prediction errors and stronger prediction abilities. The R^2^ value, ranging from 0 to 1, signifies the model’s goodness of fit, with values closer to 1 indicating better fit and closer to 0 indicating weaker predictive power.

#### Model parameter setting

The hyper-parameters of the proposed SSO–LSTM model, including the number of neurons in the first hidden layer (*h*_*t1*_), the number of neurons in the second hidden layer(*h*_*t2*_), the number of iterations (*i*_*t*_), and the learning rate (*l*_*t*_)*,* were set to [1,100], [1,100], [1,500], and [0.001,0.01], respectively*.* For the traditional LSTM model, the parameters *h*_*t1*_, *h*_*t2*_, *i*_*t*_, and *l*_*t*_, were set to 100, 100, 500 and 0.01, respectively^24^*.* The parameters of the traditional BP model, including the maximum iteration times, the learning rate, and the convergence error were set as 500, 0.01, and 0.01, respectively^16^.

#### Results analysis of trend component displacement

**Figure 7:**
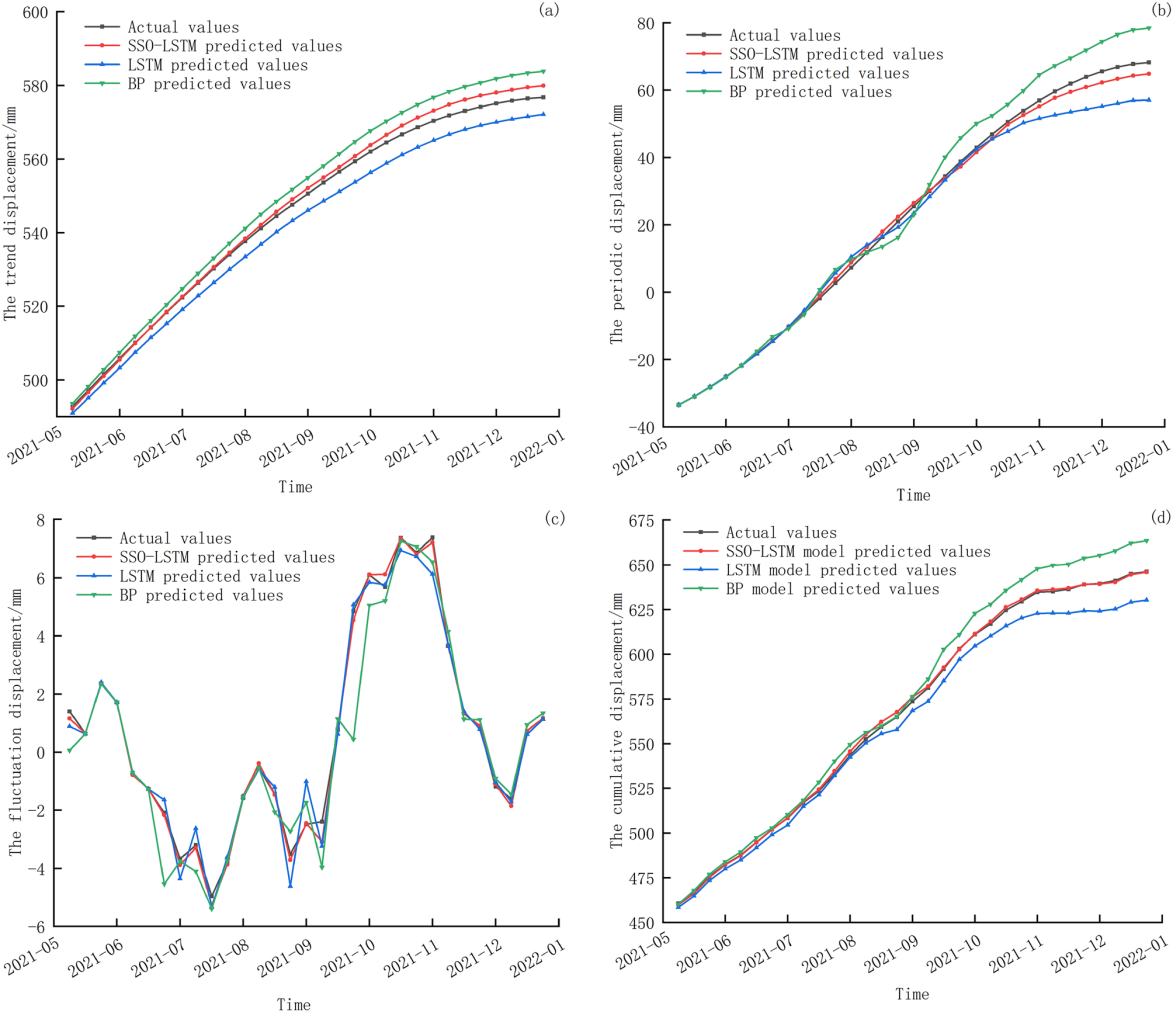
GNSS32 Prediction results of (**a**) the trend component displacement, (**b**) the periodic component displacement, (**c**) the fluctuation component displacement, and (**d**) the cumulative displacement.

The displacement of the trend component is mainly influenced by geological factors within the landslide. To predict trend component displacement, a rolling prediction method is employed: the trend component displacement from the previous 4 weeks is used as input to predict the trend displacement for the next week. Figure 7a illustrates the prediction results of trend component displacement. The displacement change of the trend term appears relatively stable, and the prediction results of the three models better reflect the movement trend of the monitoring curve without significant fluctuation. However, the prediction curve of the SSO–LSTM model is closer to the actual value curve, indicating a superior prediction effect. Table 3 provides a clear comparison, showing that the error values predicted by the LSTM model and BP model are similar, while the error values predicted by the SSO–LSTM model are slightly lower.

**Table 3:** GNSS32 error analysis of displacement component prediction results.

	SSO–LSTM			LSTM			BP		
	RMSE (mm)	MAPE (%)	R^2^	RMSE (mm)	MAPE (%)	R^2^	RMSE (mm)	MAPE (%)	R^2^
Trend component	0.9551	0.2633	0.9951	4.4169	0.7743	0.9723	4.7336	0.7684	0.9678
Periodic component	1.6993	6.4212	0.9975	4.9844	14.8993	0.9787	5.4181	19.2305	0.9748
Fluctuation component	0.1952	5.2254	0.9968	0.5206	13.5734	0.9977	1.0573	20.6493	0.9063
Cumulative displacement	1.2329	0.1624	0.9969	8.4887	1.141	0.9811	10.6634	1.4839	0.9656

#### Results analysis of periodic component displacement

Based on the analysis above, the displacement of the periodic component is primarily influenced by rainfall. The input variables for the SSO–LSTM model consist of the low frequency component of accumulated rainfall in the current week, the previous week and the previous 2 weeks, along with the periodic displacement component during the same time period. The output value is the landslide displacement for the following week. The prediction results and comparison of periodic component displacement are presented in Fig. 7b and Table 3. The findings are as follows: (1) Both the traditional LSTM and the proposed SSO–LSTM models demonstrate a good ability to track the periodic displacement components. The traditional BP model initially tracks the displacement change trend well, but after July 2021, its prediction accuracy gradually deteriorates, with the prediction curve fluctuating sharply around the monitoring curve. (2) The Mean Absolute Percentage Error (MAPE) value of the SSO–LSTM model is obviously smaller than the other two models, and the prediction effect is better.

#### Results analysis fluctuation component displacement

The fluctuation displacement is primarily affected by external random factors such as non-seasonal rainfall. When high frequency rainfall is combined to predict the fluctuation displacement component, SSO–LSTM input variables include the high frequency component of accumulated rainfall in the current week, the previous week and the previous 2 weeks, and the fluctuation displacement component of the same period. The prediction results of fluctuation component displacement are shown in Fig. 7c and summarized in Table 3. Key observations are as follows: (1) The displacement monitoring curve fluctuates sharply from June to October 2021, leading to a sharp decline in the predictive ability of the BP mode. As the model's error adjustment ability deteriorates, the loss of detailed information results in the largest prediction error in September 2021. Although the prediction curves of the LSTM model and SSO–LSTM model fluctuate around the fluctuation displacement monitoring curve, they capture the overall motion trend of the fluctuation term displacement. (2) The R^2^ value predicted by the BP model, which exhibits high volatility, is only 0.9063, indicating poor fitting. In contrast, the R^2^ value predicted by the LSTM model and the SSO–LSTM model is close to 1, indicating that those two LSTM models are more suitable for the predicting of the displacement curve with obvious dynamic characteristics. The proposed SSO–LSTM model achieves an RSEM value of 0.1952 and MAPE value of 5.2254, indicating the smallest error in predicted values and the best prediction effect for the dynamic prediction model.

#### Results analysis of landslide cumulative displacement

The cumulative displacement was obtained by combining the three predicted displacement components, as depicted in Fig. 7d and summarized in Table 3. The prediction graph reveals the inadequacy tracking ability of the BP model, and its poor adaptability to the wave displacement trend when external disturbance increase. In contrast, among the three models, the prediction curve of the proposed SSO–LSTM model fits the monitoring curve most well. As shown in Table 4, compared with the LSTM model, the RMSE and MAPE values of the proposed SSO–LSTM dynamic prediction model decreased by 85.48% and 85.77%, respectively. Compared with the BP model, the RMSE and MAPE values of the SSO–LSTM model were reduced by 88.44% and 88.26%, respectively.

**Table 4:** Comparison of GNSS32 prediction errors between with and without VMD processing.

	SSO–LSTM			LSTM			BP		
	RMSE (mm)	MAPE (%)	R^2^	RMSE (mm)	MAPE (%)	R^2^	RMSE (mm)	MAPE (%)	R^2^
With VMD	1.2329	0.1624	0.9969	8.4887	1.141	0.9811	10.6634	1.4839	0.9656
Without VMD	1.8827	0.9574	0.9685	12.8554	1.7408	0.9567	16.1454	2.0027	0.9317

#### Analysis of the impact of signal VMD decomposition and reconstruction on prediction accuracy

**Figure 8:**
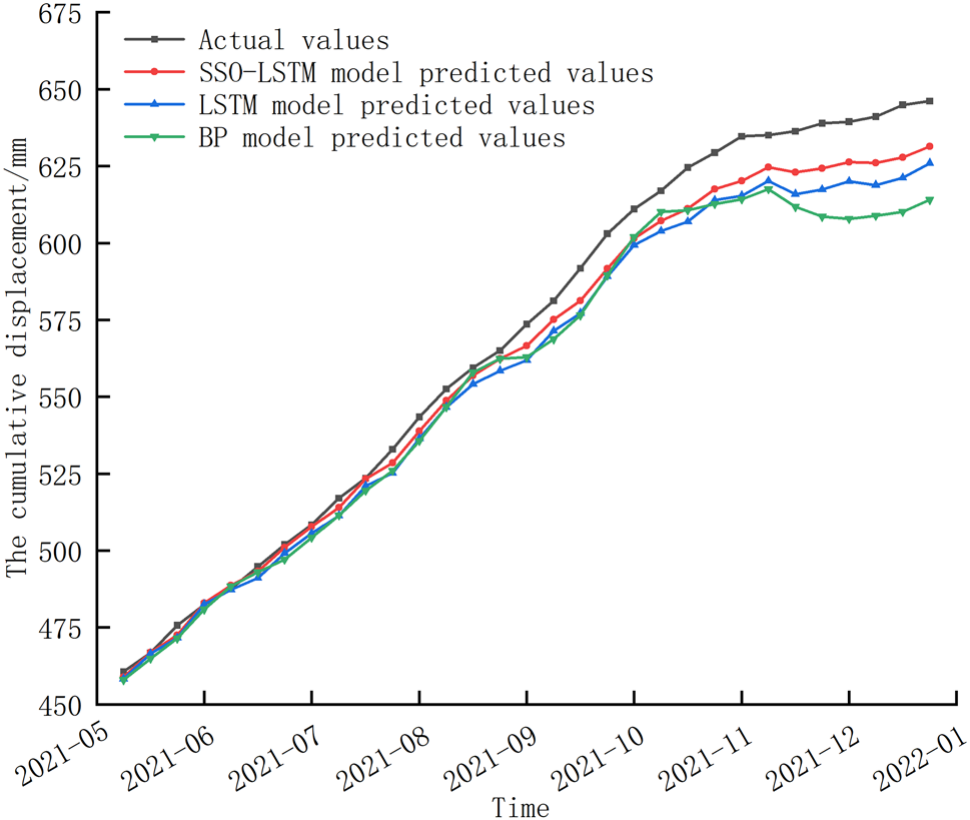
Comparison of GNSS32 prediction results using the original cumulative displacement without VMD processing.

To assess the impact of Variational Mode Decomposition (VMD) decomposition and reconstruction on prediction accuracy, we conducted a comparative analysis of the three models using the original cumulative displacement data set without VMD processing, as shown in Fig. 8. The prediction errors were compared with those obtained using VMD processing, as summarized in Table 4.

Figure 8 illustrates that the prediction curves of the three models can generally track the movement trend of the monitoring curve, but their adjustment ability is limited. The deviation between the prediction curve and the monitoring curve gradually increases as the prediction time progresses, with the error peaking at the end of the prediction period. Table 4 revealed that the incorporation of VMD decomposition and reconstruction significantly improved the prediction accuracy of the SSO–LSTM model. Specifically, the RMSE and MAPE values were reduced by 34.51% and 78.56%, respectively, compared to the model without VMD processing. This improvement can be attributed to the ability of VMD to effectively extract the underlying components of the signal, thereby reducing noise and improving the model's ability to capture the true underlying patterns in the data.

Overall, our findings suggest that the incorporation of VMD decomposition and reconstruction can enhance the prediction accuracy of landslide displacement models, particularly when using deep learning models such as SSO–LSTM.

## Discussions

### Discussion on considering external rainfall factors

The displacement curve of the HP21 landslide exhibits a stepped-upward change, with rapid deformation stages coinciding with heavy rainfall. During periods of low or no rainfall, landslide displacement and deformation are minimal. This indicates that both internal evolution and external rainfall factors influence landslide displacement and deformation, which is consistent with findings from Refs.^27,28^. Therefore, incorporating external rainfall as a key input parameter in the prediction model construction improves the model's ability to track abrupt landslide displacement. Compared to direct prediction of landslide displacement, the model's predicted values align more closely with measured values. The root means square error (RMSE), mean absolute percentage error (MAPE), and goodness of fit (R^2^) for the predicted cumulative displacement were 1.2329, 0.1624%, and 0.9969, respectively.

### Discussion on ADF test of VMD

The traditional VMD method typically sets the mode number, K, to 3 to decompose landslide cumulative displacement^10^. However, this approach often leads to suboptimal decomposition results, with the Intrinsic Mode Function (IMF) sequence containing multiple displacement information. In this study, the Augmented Dickey–Fuller (ADF) test was employed to determine the optimal number of VMD decomposition layers, resulting in 5 IMF components. Subsequently, based on the fluctuation characteristics and smoothness of each IMF component, the trend term displacement, periodic term displacement, and fluctuation term displacement—each with clear physical significance—were reconstructed. The predictions for the three displacement components were combined to obtain the predicted accumulated displacement. Compared to prediction results without VMD processing, the cumulative displacement prediction exhibiting a more stable movement trend and improved anti-interference ability in prediction.

### Discussion on the applicability the proposed VMD–SSO–LSTM model

The LSTM model can utilize and retain historical information, leveraging its capability to extract correlation information from past and future time series data, thereby enhancing displacement prediction accuracy. Compared to the static BP model, the dynamic LSTM model demonstrates higher prediction accuracy on the same training set. The SSO algorithm's adaptive iterative search for LSTM model hyper-parameters ensures rapid and stable convergence to the global optimal solution in the multidimensional hyper-parameter space, significantly enhancing model performance.

The trained VMD–SSO–LSTM model was employed to forecast the monitoring data of GNSS28 monitoring points for a 3-month period, with error analysis conducted on the prediction results, as shown in Table 5. the RSEM, MAPE, and R^2^ values of are 1.5969, 0.4266%, and 0.9971, respectively. The prediction results indicate that the VMD–SSO–LSTM model exhibits good predictive performance when forecasting short-term monitoring data, demonstrating high applicability and strong stability.

**Table 5:** Comparison of GNSS28 prediction results by using a 3-month VMD processing data set.

Model type	RMSE (mm)	MAPE (%)	R^2^
SSO–LSTM	1.5969	0.4266	0.9971
LSTM	4.9171	1.3495	0.9725
BP	8.0727	1.8074	0.9258

### Discussion on considering stochastic factors

In the realm of displacement prediction research, constrained by current monitoring methods, this study solely contemplates the impact of rainfall and the inherent evolution stage of landslides on the stochastic displacement component. The study overlooks the influence of stochastic factors such as artificial loads. Nevertheless, stochastic displacement embodies a comprehensive reflection of natural environmental variations, rendering precise prediction of stochastic displacement imperative for landslides triggered by external environmental changes.

## Conclusions

Aiming at the complex nonlinear system of landslide, a dynamic prediction model of landslide displacement based on VMD–SSO–LSTM is proposed, and the model is analyzed and verified by the actual monitoring data of active landslide. The following conclusions are obtained: (1) The VMD method can effectively reduce interfering data in landslide cumulative displacement time series signals. By decomposing the signals and reconstructing the components, displacement components with clear physical meaning can be obtained, which better explain the changes in landslide displacement. (2) The LSTM model can accurately memorize and predict the historical information of monitoring points. The sparrow search algorithm can efficiently, stably, and adaptively find the optimal hyperparameters of the LSTM model. Finally, a prediction model is developed to improve the accuracy of dynamic prediction of landslide displacements. (3) The prediction results of GNSS32 and GNSS28 monitoring points show that compared with the traditional LSTM model and BP model, the proposed VMD–SSO–LSTM model has good accuracy.

To further enhance the prediction performance of the model, it is recommended to incorporate additional parameters closely linked to landslide stability, such as soil sliding resistance. Moreover, converting the landslide displacement prediction results into the probability of landslide occurrence can further enhance decision-making capabilities.

## Data Availability

The data used to support the f**i**ndings of this study are available from the corresponding author upon request.
